# A Two-To-One Deep Learning General Framework for Image Fusion

**DOI:** 10.3389/fbioe.2022.923364

**Published:** 2022-07-14

**Authors:** Pan Zhu, Wanqi Ouyang, Yongxing Guo, Xinglin Zhou

**Affiliations:** ^1^ Key Laboratory of Metallurgical Equipment and Control Technology, Ministry of Education, Wuhan University of Science and Technology, Wuhan, China; ^2^ Hubei Key Laboratory of Mechanical Transmission and Manufacturing Engineering, Wuhan University of Science and Technology, Wuhan, China; ^3^ Precision Manufacturing Institute, Wuhan University of Science and Technology, Wuhan, China

**Keywords:** bionic vision, multi-modal image fusion, convolutional neural network, y-distribution structure, multi-convolution kernel, adaptive feature analysis

## Abstract

The image fusion algorithm has great application value in the domain of computer vision, which makes the fused image have a more comprehensive and clearer description of the scene, and is beneficial to human eye recognition and automatic mechanical detection. In recent years, image fusion algorithms have achieved great success in different domains. However, it still has huge challenges in terms of the generalization of multi-modal image fusion. In reaction to this problem, this paper proposes a general image fusion framework based on an improved convolutional neural network. Firstly, the feature information of the input image is captured by the multiple feature extraction layers, and then multiple feature maps are stacked along the number of channels to acquire the feature fusion map. Finally, feature maps, which are derived from multiple feature extraction layers, are stacked in high dimensions by skip connection and convolution filtering for reconstruction to produce the final result. In this paper, multi-modal images are gained from multiple datasets to produce a large sample space to adequately train the network. Compared with the existing convolutional neural networks and traditional fusion algorithms, the proposed model not only has generality and stability but also has some strengths in subjective visualization and objective evaluation, while the average running time is at least 94% faster than the reference algorithm based on neural network.

## 1 Introduction

Deep learning is a bio-inspired intelligent computing technology that is based on the principles of neurotransmission processes in the human brain, which resembles the pattern of connections between brain neurons (Xu et al., 2021). Unlike classical bionic techniques, i. e., ant colony algorithms (Deng et al., 2020), bee algorithms (Çil et al., 2020), etc., and particle swarm optimization (Elbes et al., 2019), etc., deep learning has an incredible and impressive ability to resolve the complexity of real-world problems, which has caused the attention of many scholars and has been successfully applied to practical problems (Chen et al., 2021b; Chen et al., 2022a; Chen et al., 2022c; Sun et al., 2022). In recent years, deep learning, especially neural networks, has become one of the most rapidly growing and widely applied artificial intelligence technologies. Several studies have demonstrated the superior performance of neural networks in target detection (Jiang et al., 2021a; Huang et al., 2021; Huang et al., 2022), image segmentation (Jiang et al., 2021b), data processing (Chen et al., 2021a; Chen et al., 2022b), and depth estimation (Jiang et al., 2019), etc. In addition, image fusion, which is an essential branch of neural network research, has been extensively implemented in various areas, especially in civil, military, and industrial applications, since the research on neural networks has gradually advanced. For example, mobile phones often integrate with high dynamic range (Ma et al., 2015; Liu et al., 2018; Qi et al., 2021) or refocus algorithms (Saha et al., 2013; Bai et al., 2015; Zhang and Levine, 2016) to get stable and information-rich images. Visible and infrared image fusion can provide a more direct monitoring environment to the observers (Xue and Blum, 2003; Wan et al., 2009; Zhou et al., 2016; Zhang et al., 2017).

Convolutional neural network (CNN), which is a category of neural networks, usually is superior to traditional manual feature extractors in feature extraction (Yan et al., 2017; Li et al., 2018), and the number of convolutional filters is significantly larger than traditional filters. Therefore, CNN can capture richer image details and is frequently used for image feature extraction. As such a potent tool, CNN provides new ideas and directions for research on image fusion. In general, neural networks enable to excavate of implicit rules in massive datasets and then predict the result by the gained rules, which render the models with exceptional generalization ability (Cheng et al., 2021; Huang et al., 2021). For traditional image fusion algorithms, multi-modal image fusion usually implies different fusion rules and it is difficult to seek a harmonized approach. As for CNN, CNN is not fully exploited in most cases and is primarily applied for image feature extraction. Although a few fully convolutional neural networks, which don’t need to impose preprocessing and fusion rules, can automate image fused, the fusion object is specified for single-modal images. Therefore, the study of the generality of multi-modal image fusion faces a tremendous challenge.

**FIGURE 1:**
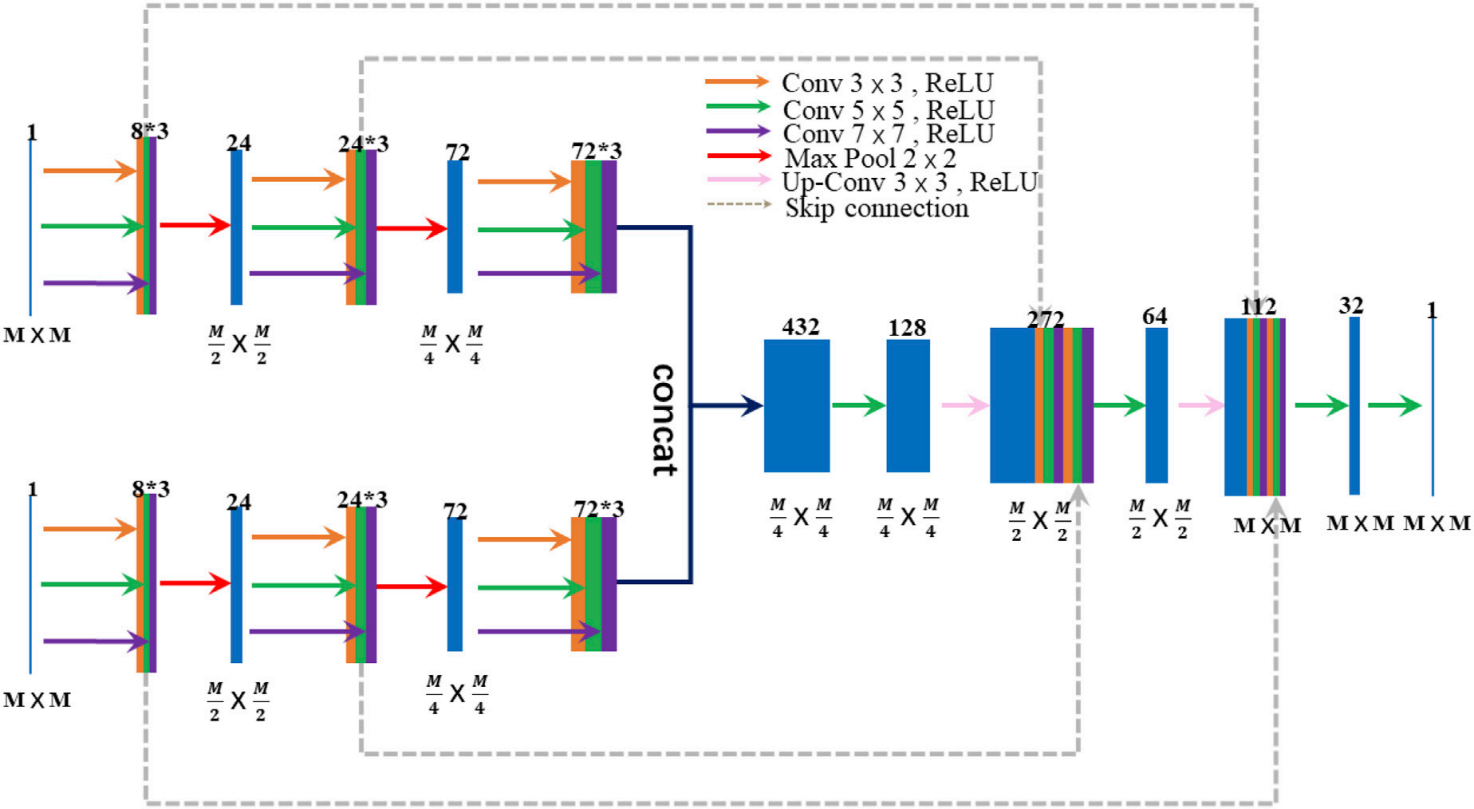
The architecture of IY-Net. M represents the size of the feature map. The number at the top block represents the feature depth.

In this paper, a general CNN framework for image fusion, called IY-Net, is designed. The structure of IY-Net is shown in Figure 1. The proposed model has two innovations. First of all, the proposed model has the characteristics of a fully convolutional neural network with relatively good generality. It doesn’t need to specify fusion rules and has a simple network structure. This is the key innovation point. Secondly, since the quality of training datasets constrains the model performance in the field of deep learning, the appropriate dataset is particularly critical. Theoretically, the performance of the model that is gained by using images of the same modal as the training dataset is more stable and accurate. However, this paper selects multi-modal images as the training dataset, and the proposed model can avoid the mutual influence of fusion results in some way. Thus, these two innovations can make the proposed model stand out from the current CNN methods.

The main contribution of this work is to propose a general image fusion framework. It is superior to many traditional algorithms and CNN methods in terms of image visual effects. The proposed model achieves excellent performance in multi-focus, infrared and visible, multi-exposure image fusion, etc. There are two more specific contributions. Firstly, a multi-feature extraction module is introduced, which effectively extends the perceptual field of the convolutional layer and thus captures more feature information. Secondly, a way of image reconstruction is constructed to effectively solve the problem of gradient disappearance and gradient explosion caused by CNN.

The rest of this paper is organized as follows. In Section 2, the paper discusses the related work. Section 3 introduces the proposed model in detail. Section 4 describes the experimental results and discusses them. In Section 5, the paper shows the conclusion and future research directions.

## 2 Related Work

Regarding CNN and traditional algorithms, despite several research results that have been achieved in image fusion algorithms, there is still space for optimization and improvement. In addition, most methods can only address image fusion of a few patterns and lack generality.

In general, traditional image fusion algorithms can be divided into two categories, i. e., spatial domain and transform domain algorithms. For image fusion algorithms in the spatial domain (Huang and Jing, 2007; Zhou et al., 2014; Zhang et al., 2017; Amin-Naji et al., 2022), the source image is divided into small pieces or regions according to certain criteria in the first step. Then the significance of the corresponding regions is evaluated, and finally, the most critical regions are fused. These algorithms are mainly applied to same-mode images, which may reduce the edge sharpness and contrast of the fused image or even produce halos at the edges. On the other hand, for the transform domain image fusion algorithm (Haghighat et al., 2011), the source image is decomposed into a feature domain by multi-scale geometry at the first step. Then, feature weighted fusion is achieved on multiple input images, and finally, the fused image is gained by the inverse transformation of the fused features. Among the current transform domain algorithms, multi-scale transform image fusion algorithms (MSTIF) are becoming increasingly popular. Examples of such transforms include pyramid-based decomposition (Liu et al., 2001), curvelet transform (Tessens et al., 2007), dual-tree complex wavelet transform (DTCWT) (Lewis et al., 2007), discrete wavelet transform (DWT) (Zheng et al., 2007; Tian and Chen, 2012) and non-subsampled contourlet transform (NSCT) (Moonon and Hu, 2015), etc. MSTIF relies on the selection of multi-scale decomposition methods and fusion strategies for multi-scale coefficient fusion. As a result, such algorithms have a relatively high manual factor, which leads to obvious weaknesses and lack of generality. For example, NSCT is weak at capturing curve details and curvelet transform is computationally complex, as well as it is terrible at multi-exposure and remote sensing image fusion. While fusing some modal images, pyramid-based decomposition will be distorted and laplace pyramid transform will incur redundant information, which is not available to infrared and visible image fusion. In conclusion, traditional MSTIF has a wide variety of filters, but it is always restricted in terms of the generality.

In recent years, image fusion methods based on neural networks have been rapidly growing (Liu et al., 2018). Firstly (Liu et al., 2017), regarded the fusion of multi-focus images as a classification task and used CNN to predict the focus image to obtain the fused image (Song et al., 2018). applied two neural networks to perform super-resolution processing of low-resolution terrestrial images and extract the feature map. Then high-pass modulation and weighting strategies are used to reconstruct the feature maps into fused images (Bhalla et al., 2022). integrated fuzzy theory with Siamese convolutional network to extract salient features of the source image as well as high-frequency information, and finally acquired fusion results by pixel strategy directly mapping to the source image. The above methods require pre-processing to generate fused images. In addition, they can only fuse images of a single-modal and lacks generality (Zhang et al., 2020). proposed a CNN-based image fusion framework that is trained in an end-to-end manner, and the parameters of the model can be jointly optimized without any subsequent processing. Although they designed a generalized model, it adopted human-selected fusion rules in the feature fusion phase, which led to the degradation of the model generality and the image fusion performance. For example, when infrared and visible images are fused, the model applies MAX fusion features to yield the best result. But when multi-exposure images are fused, it employs SUM fusion features to gain the best result. In summary, although CNN has achieved some success in the domain of image fusion, the majority of current models lack generality. In addition, most CNN is not designed end-to-end (Wang et al., 2019a) and requires additional steps to complete the task. Therefore, the CNN-based image fusion model has not been fully exploited, and there is still much potential to be boosted in terms of generality.

## 3 Methods and Materials

### 3.1 Feature Extraction Module

The convolutional layer in CNN extracts different feature information from the training image by convolutional kernels and then updates the filter parameters automatically. Therefore, the selection of convolutional kernels is crucial for feature extraction. The specific structure is shown in Supplementary Figure S1. The small-size convolution kernel is used to extract the low-frequency and small detail information, while high-frequency and large detail information can’t be detected. Likewise, the large size of the convolution kernel is preferable for identifying high-frequency and large detail information.

**FIGURE 2:**
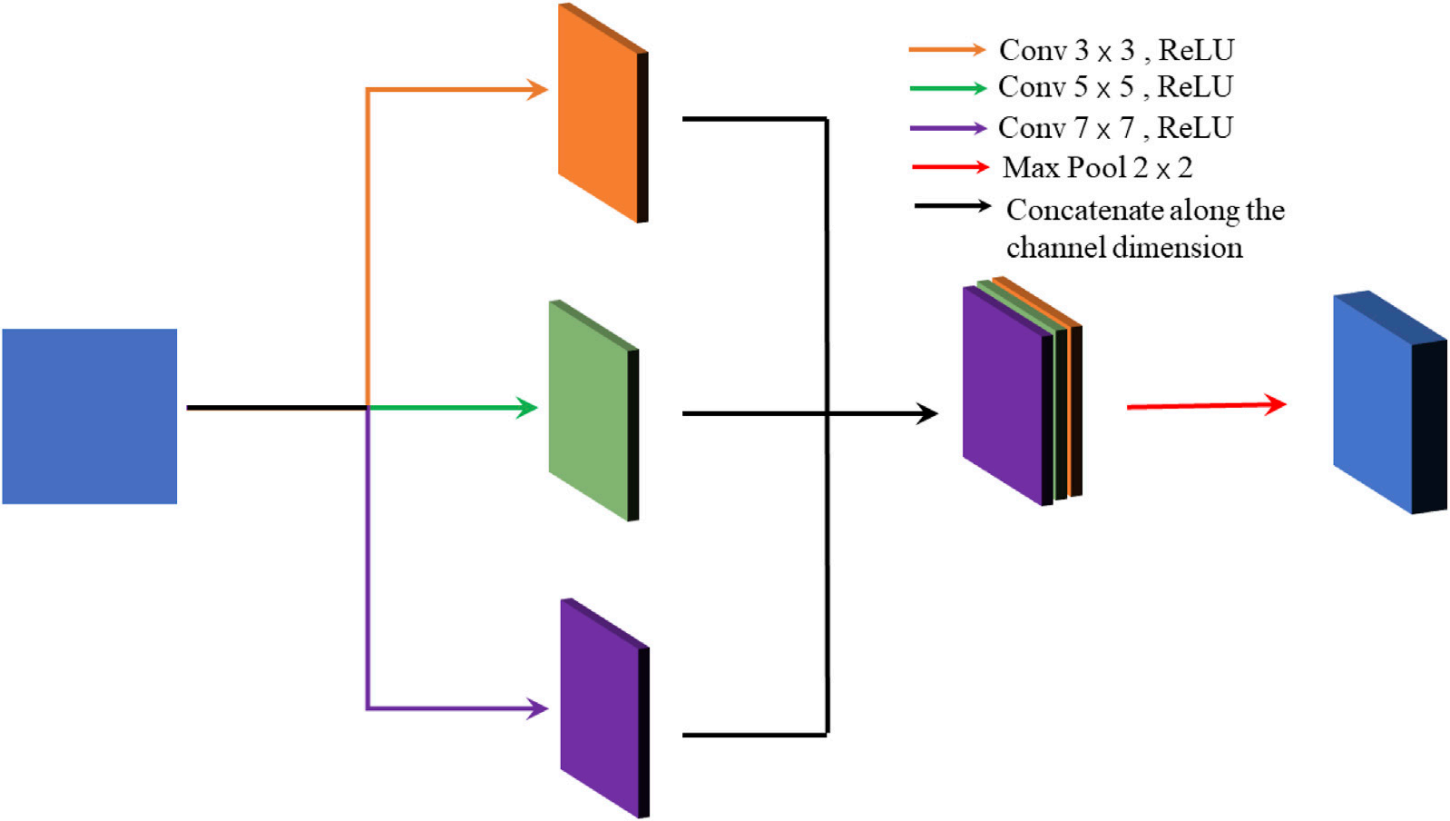
Structure of multi-feature extraction layer.

As stated above, the paper utilizes multiple feature extraction layers, each of which has convolution kernels of sizes 3 × 3, 5 × 5, and 7 × 7, to capture low and high-frequency information. The specific structure is shown in Figure 2. The proposed model detects the feature information of the input image by three multiple feature extraction layers, but multiple convolutions can lead to over-fitting and increasing the training time. Therefore, this paper adds a max-pooling layer after both of the two previous multiple feature extraction layers to avoid such phenomena.

### 3.2 Feature Fusion Module

**FIGURE 3:**
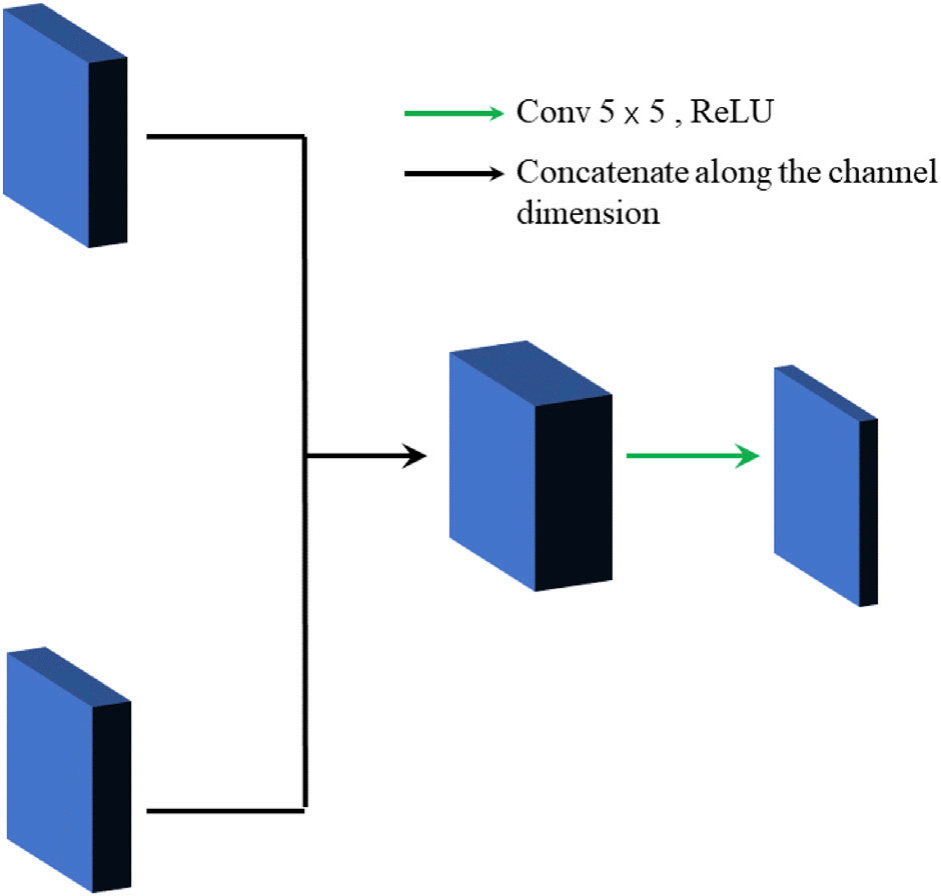
Feature fusion structure.

There are two general methods for feature fusion: 1) The feature maps are connected along with the number of channels. 2) The feature maps are fused according to certain fusion rules. If the second feature fusion way is chosen, it will lead to a decrease in the generality of the model. Therefore, the paper chooses the first method to get the fused feature map. The specific structure is shown in Figure 3. Firstly, the feature maps are concatenated along the channel dimension to gain the initial feature fusion map, and then it is filtered by the convolution layer. Finally, it is down-dimensioned to produce the final cross-channel fused feature map.

### 3.3 Image Reconstruction Module

**FIGURE 4:**
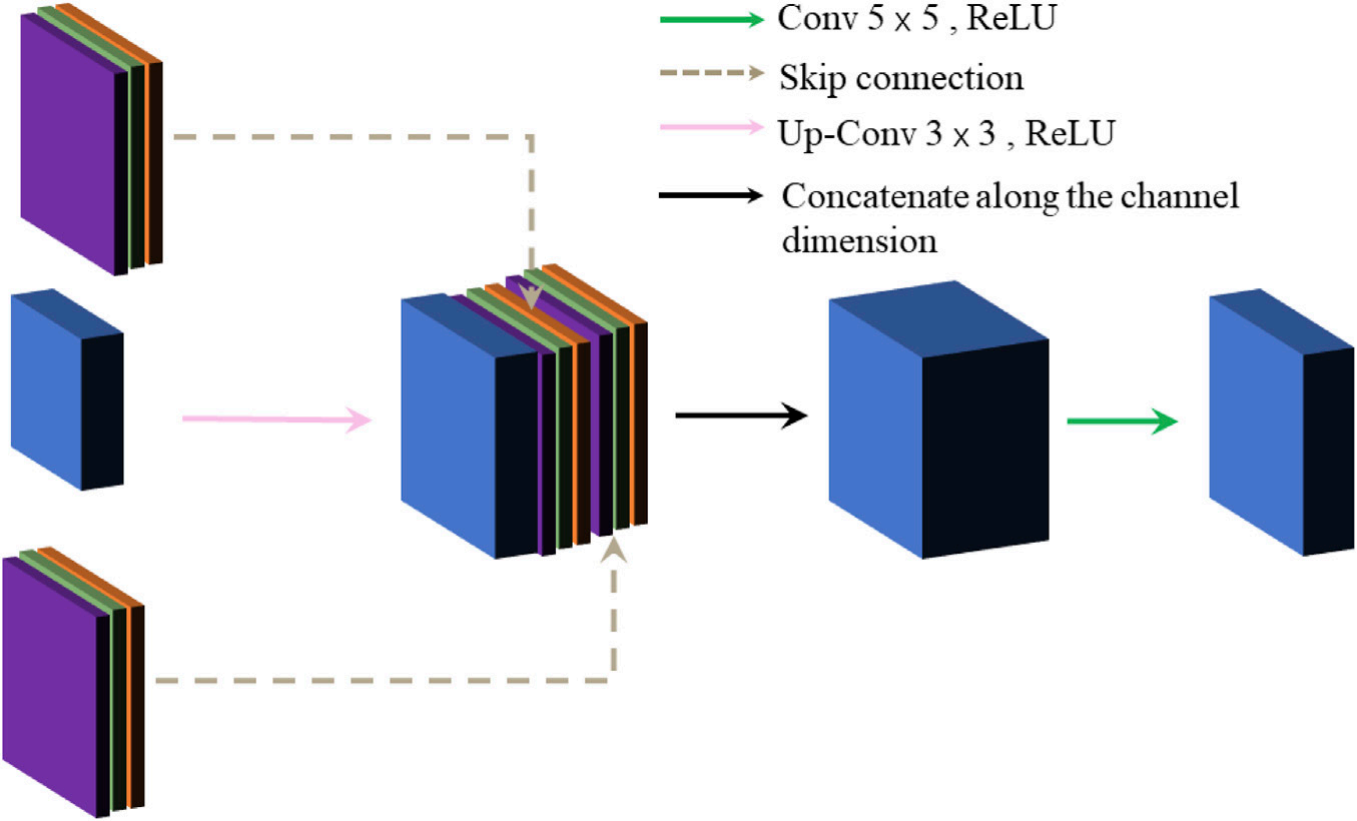
Up-sampling operations and skip connection structure.

Under the effect of the pooling layer, the image size is changed from 256 × 256 to 64 × 64, which greatly reduces the resolution of the original image, and some features may be ambiguous. For restoring the size of the resource image, the paper applies the up-sampling operation (i.e., transposed convolution) to restore the resolution and optimize the image quality. However, it causes the image edge information to be dropped and blurred, so we deal with this problem by adding a skip connection based on the up-sampling operation, which can further enhance the image edge information. The module undergoes three up-sampling operations, which each time doubles the image size, and eventually produces a grayscale image with the original size. The specific up-sampling operations and skip connection structure are shown in Figure 4. Firstly, the feature map and the fused feature map are skip-connected, and then up-sampling operations are executed on them. Finally, the high-dimensional map is down-dimensioned to a low-dimensional map by convolutional layers.

### 3.4 Loss Function

Before training the model, it is necessary to optimize the model parameters using an appropriate loss function to compare the predicted values with the actual values. The proposed model aims to form a fused image by regression of two input images. Therefore, the paper chooses the structural similarity (SSIM) (Wang et al., 2004) to coping with this problem. As shown in the equation.

$$\mathrm{SSIM}\left(x,y\right)=\frac{\left(2\mu_{x}\mu_{y}+C_{1}\right)\left(2\sigma_{\mathrm{xy}}+C_{2}\right)}{\left(\mu_{x}^{2}+\mu_{y}^{2}+C_{1}\right)\left(\sigma_{x}^{2}+\sigma_{y}^{2}+C_{2}\right)}$$ (1)

Where *x* is the real image, *y* is the predicted image, $\mu_{x}$ , $\mu_{y}$ is mean, $\sigma_{x}$ , $\sigma_{y}$ is variance, and $\sigma_{xy}$ is covariance. $C_{1}={\left(Lk_{1}\right)}^{2}$ , $C_{2}={\left(Lk_{2}\right)}^{2}$ are stable constants. *L* is the dynamic range of pixel values, $k_{1}=0.01$ , $k_{2}=0.03$ . The sliding window size is set as 11×11 , it moves pixel by pixel in an image from top-left on an image.

Thus, SSIM loss function can be defined as:

$$L_{\mathrm{ssim}}=\frac{1}{n}\sum 1-\mathrm{SSIM}\left(x,y\right)$$ (2)

Where *n* represents the total number of sliding windows.

The proposed model has all components of the loss function that are differentiable, thus the model parameters of the paper can be updated by random gradient descent and back-propagation.

### 3.5 Training Dataset

It is well known that CNNs are data-driven. So large-scale image datasets are the basis for achieving favorable performance (Liu et al., 2017). randomly selected multi-focus images from the ImageNet dataset. And the focused images were obscured with a random scale of the Gaussian kernel to generate an image dataset consisting of 2 million pairs of images of size 16 × 16. Since no large-scale multi-exposure image dataset was available (Ram Prabhakar et al., 2017), randomly cropped 64 × 64 image segments from small multi-exposure images to generate a multi-exposure dataset.

As mentioned above, current experimental objects are composed mainly of small blocks of images as single-modal datasets, which can’t fulfill the experimental requirements. Therefore, multi-focus images, multi-exposure images, and remote sensing images are selected from several datasets to form the training dataset with an image size of 256 × 256 in this paper. The images in the training dataset was are randomly rotated, randomly contrast shifted, and randomly stretched to boost diversity. The parts of multi-modal images in the dataset are shown in Supplementary Figure S2 **.**

## 4 Experiments and Results

### 4.1 Experimental Settings

IY-Net is implemented by Pytorch 1.8.1 based on Python 3.9.4. The proposed model is trained and tested on a computer equipped with an Intel i5-1035G1 CPU (1 GHz) and 2 GB GPU, and it is trained on the CPU. The paper trains 1826 pairs of images with an image size of 256 × 256 and a batch size of 40 in the training process. The whole process takes about 1 h. Concerning the learning rate, using the Adam optimizer (Wang et al., 2004) and the learning rate set to 0.0005.

In this paper, the proposed model is compared with traditional multi-scale transform algorithms, i. e., discrete wavelet transforms (DWT) (Zheng et al., 2007) and non-subsampled contourlet transform (NSCT) (Moonon and Hu, 2015). To further validate the advantages of the proposed model in the area of deep learning, it is compared with three current neural network-based image fusion models, i. e., multi-focus image fusion model (MFCNN) (Liu et al., 2017), CNN integration model for image fusion (ECNN) (Amin-Naji et al., 2019) and unsupervised depth model for image fusion (SESF) (Ma et al. 2021). To verify the generality of the proposed model, five types of datasets (including multifocal images, infrared and visual images, etc.) are experimented and evaluated in the paper. The five image test datasets are shown in Supplementary Figures S7,S8,S9,S10,S11.

For the evaluation of the image fusion algorithm, the paper qualitatively judges the visual effect of the fused images. The performance of different image fusion methods can’t be distinguished by visual effects alone. Therefore, five metrics are introduced to further estimate the quantitative manifestation of IY-Net on multi-modal image fusion. The five metrics are spatial frequency (SF), information entropy (IE), average gradient (AG) (Petrović, 2007), Peille index (Peille) (Piella and Heijmans, 2003), and edge preservation information (Q_AB_) (Xydeas and Petrovic, 2000) respectively.

### 4.2 Experimental Results and Analysis

#### 4.2.1 Multi-Focus Image Fusion

**FIGURE 5:**
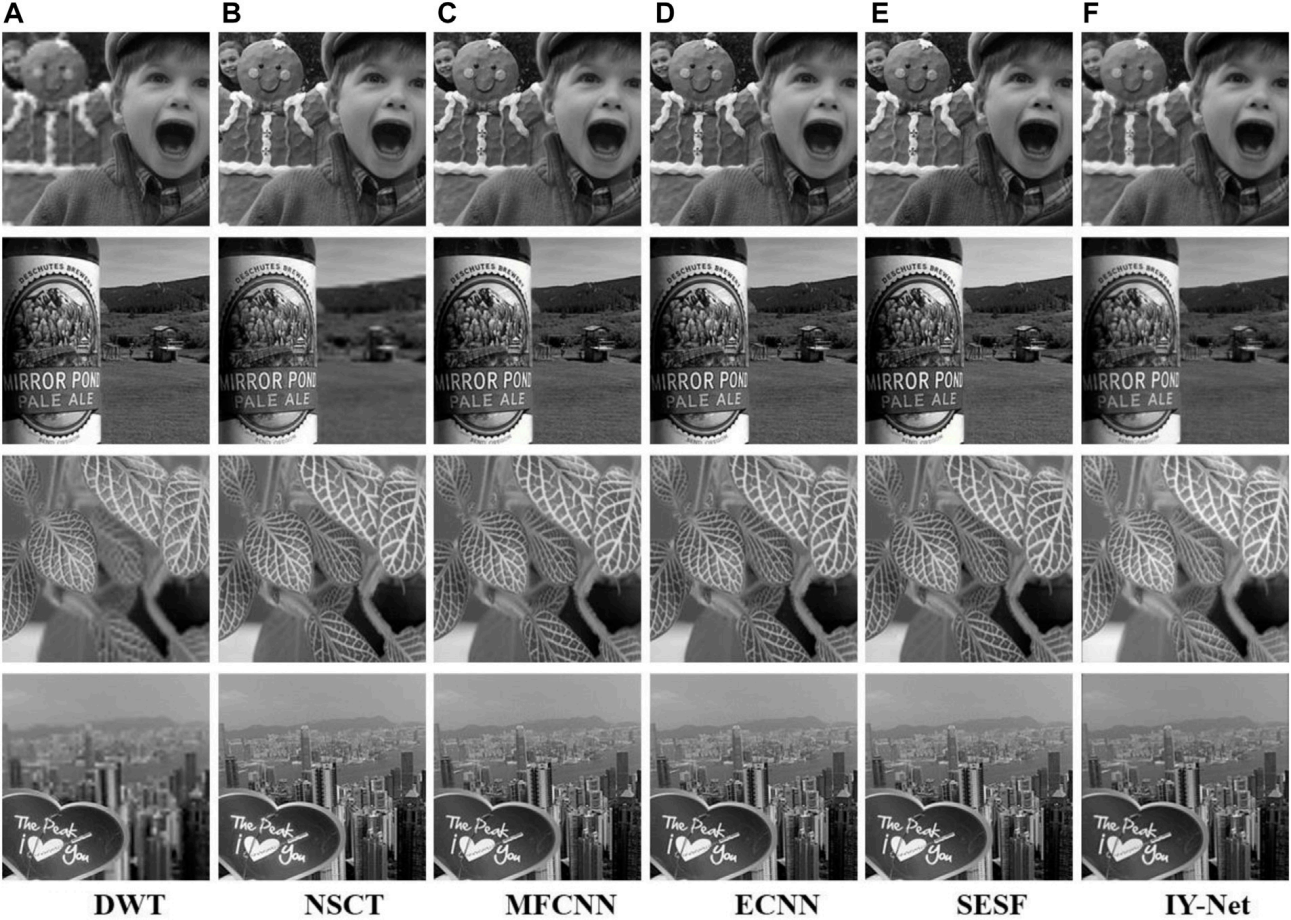
Experiment on 4 pairs of multi-focus images. **(A)** DWT, **(B)** NSCT, **(C)** MFCNN, **(D)** ECNN, **(E)** SESF, **(F)** IY-Net.

Experiments are conducted on multi-focus image test datasets as shown in Supplementary Figure S3. It is verified that the proposed model has a great performance in multi-focus image fusion. Taking “Boy” as shown in Supplementary Figure S8 (A) and (B) for example. The fusion result of DWT is blurred in some regions and fails to retain the complete details and features, but other algorithms can capture suitable feature information with better visual effects. Figure 5 provides the fusion results of multi-focus image test datasets based on all algorithms. Experimental results show that the proposed model is practicable and stable in multi-focus image fusion visually.

#### 4.2.2 Infrared and Visible Image Fusion

**FIGURE 6:**
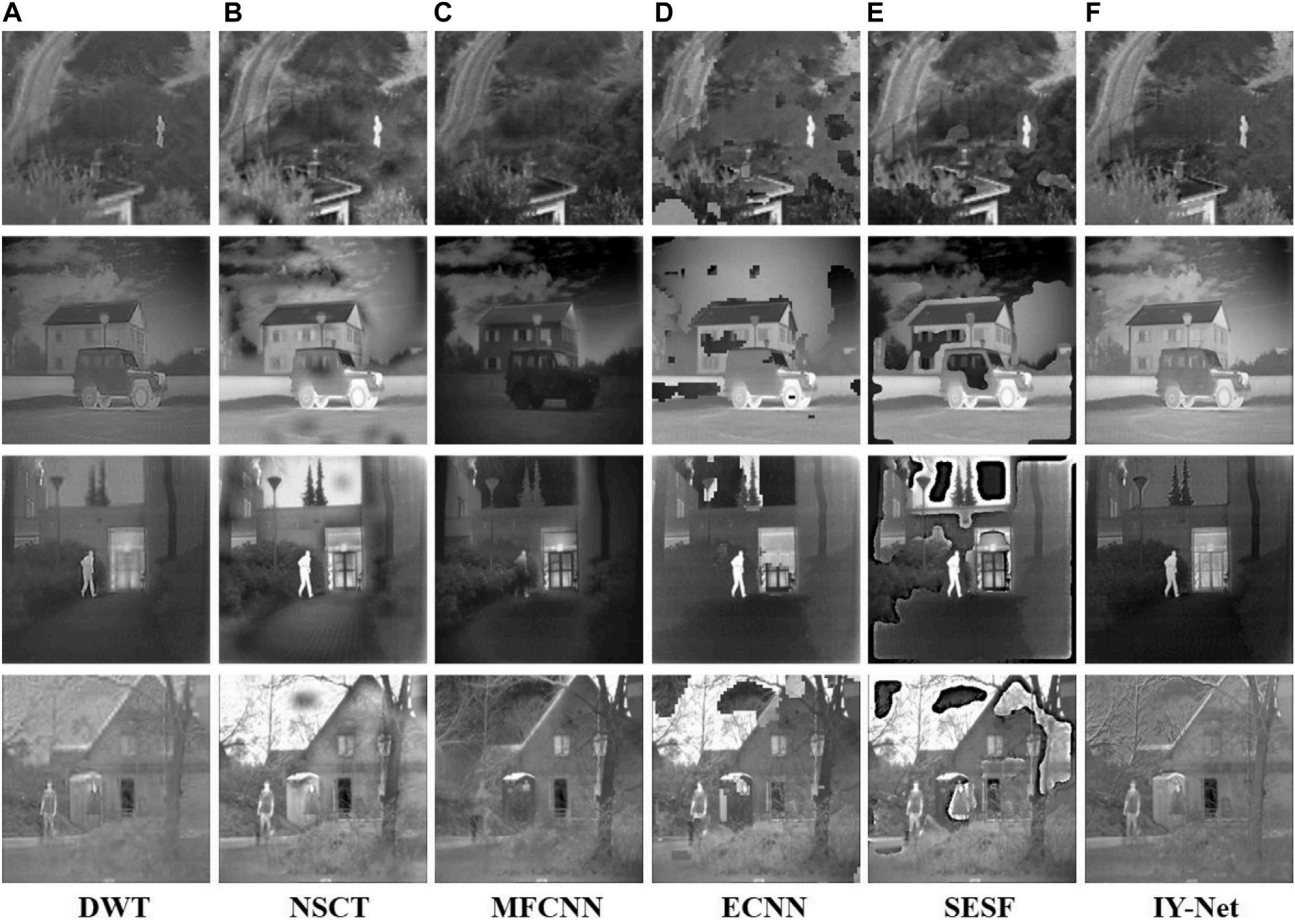
Experiment on 4 pairs of infrared and visible images. **(A)** DWT, **(B)** NSCT, **(C)** MFCNN, **(D)** ECNN, **(E)** SESF, **(F)** IY-Net.

As shown in Supplementary Figure S4, four groups of infrared and visible images reveal different scene information. Experiments are carried on them to confirm the capability of IY-Net in infrared and visible image fusion. For simplicity, “Car” is used for detailed analysis in Supplementary Figure S9. Apparently, DWT basically preserves the infrared and visible features, but the fused image has relatively low contrast. MFCNN failed to capture the infrared features and the visual effect is weak. NSCT, ECNN, and SESF produce large areas of dark spots and shadows that generate no-desired results. Exhilaratingly, IY-Net acquires the most observable fusion results, which be provided with abundant visible details and infrared features as shown in Supplementary Figure S9 (H). A similar situation occurs in Figure 6 which is obtained from the images in Supplementary Figure S4. To all appearances, IY-Net not only has the best visual effect but also possesses evident stability and adaptability in infrared and visible image fusion.

#### 4.2.3 Infrared Intensity and Polarization Image Fusion

**FIGURE 7:**
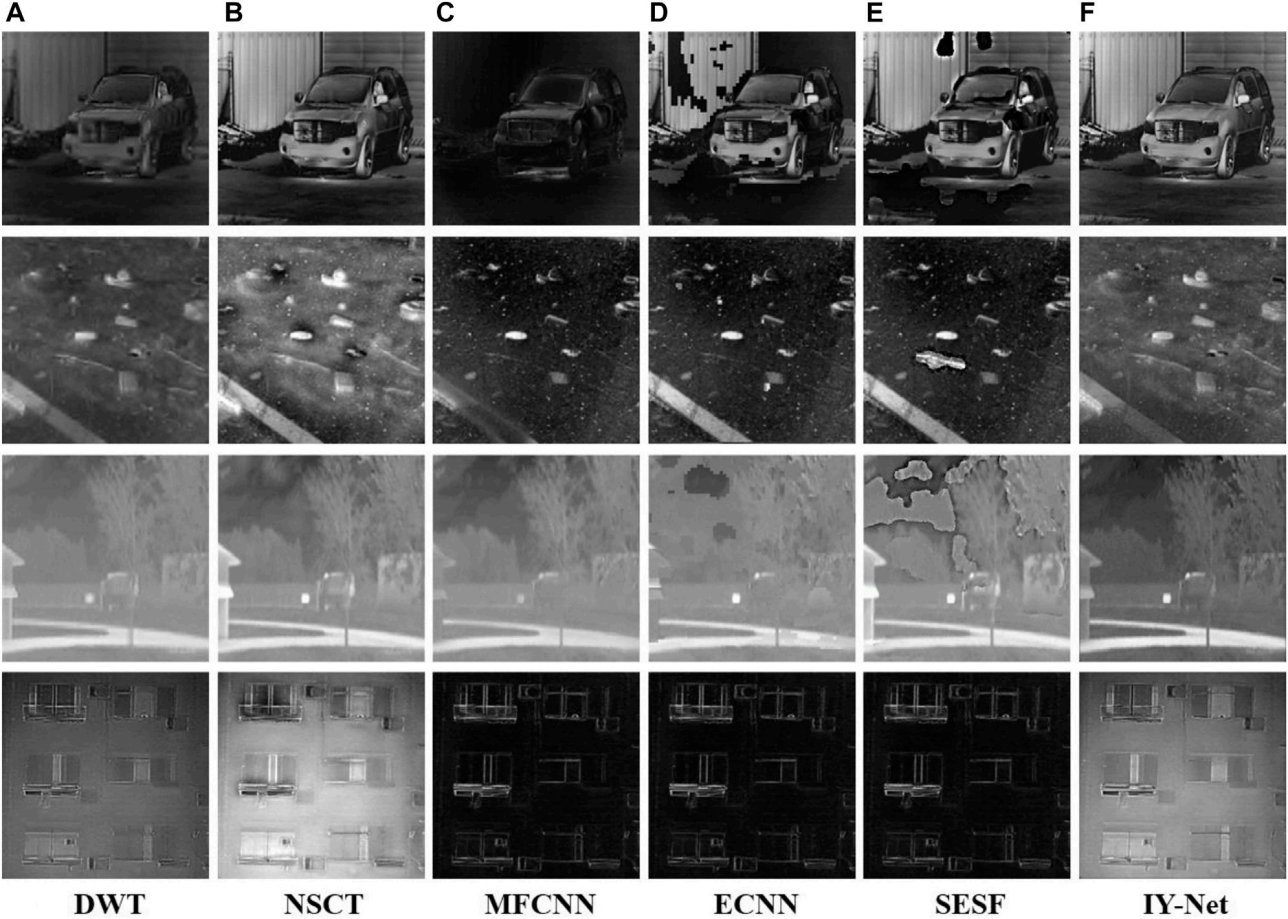
Experiment on 4 pairs of infrared intensity and polarization images. **(A)** DWT, **(B)** NSCT, **(C)** MFCNN, **(D)** ECNN, **(E)** SESF, **(F)** IY-Net.

Supplementary Figure S5 shows four pairs of infrared intensity and polarization images that are used to check the performance of the proposed model. A group of experimental results, taking “SUV” for example, is presented in Supplementary Figure S10. The source polarization and infrared intensity images are shown in Supplementary Figure S10. From the results of the experiment, we can see that DWT may maintain polarization and intensity information, but some parts are obscured, which results in poor visual effects. MFCNN cannot fuse the source image validly at all. ECNN and SESF can only combine the polarization and intensity information in part of the region and generate many pixel blocks and black spots, which seriously affects overall visual perception. In contrast, IY-Net and NSCT perfectly integrate these two kinds of images. It shows that NSCT and IY-Net could be employed availably in infrared intensity and polarization image fusion compared to other algorithms. The other fusion results are shown in Figure 7 Experiments demonstrated that MFCNN, ECNN, and SESF failed to fuse infrared intensity and polarization images in a dark environment. In addition, it produces the phenomenon of image distortion and partial texture being blurred in bright environments. However, NSCT and IY-Net can be adapted for infrared intensity and polarization image fusion in different environments.

#### 4.2.4 Multi-Exposure Image Fusion

**FIGURE 8:**
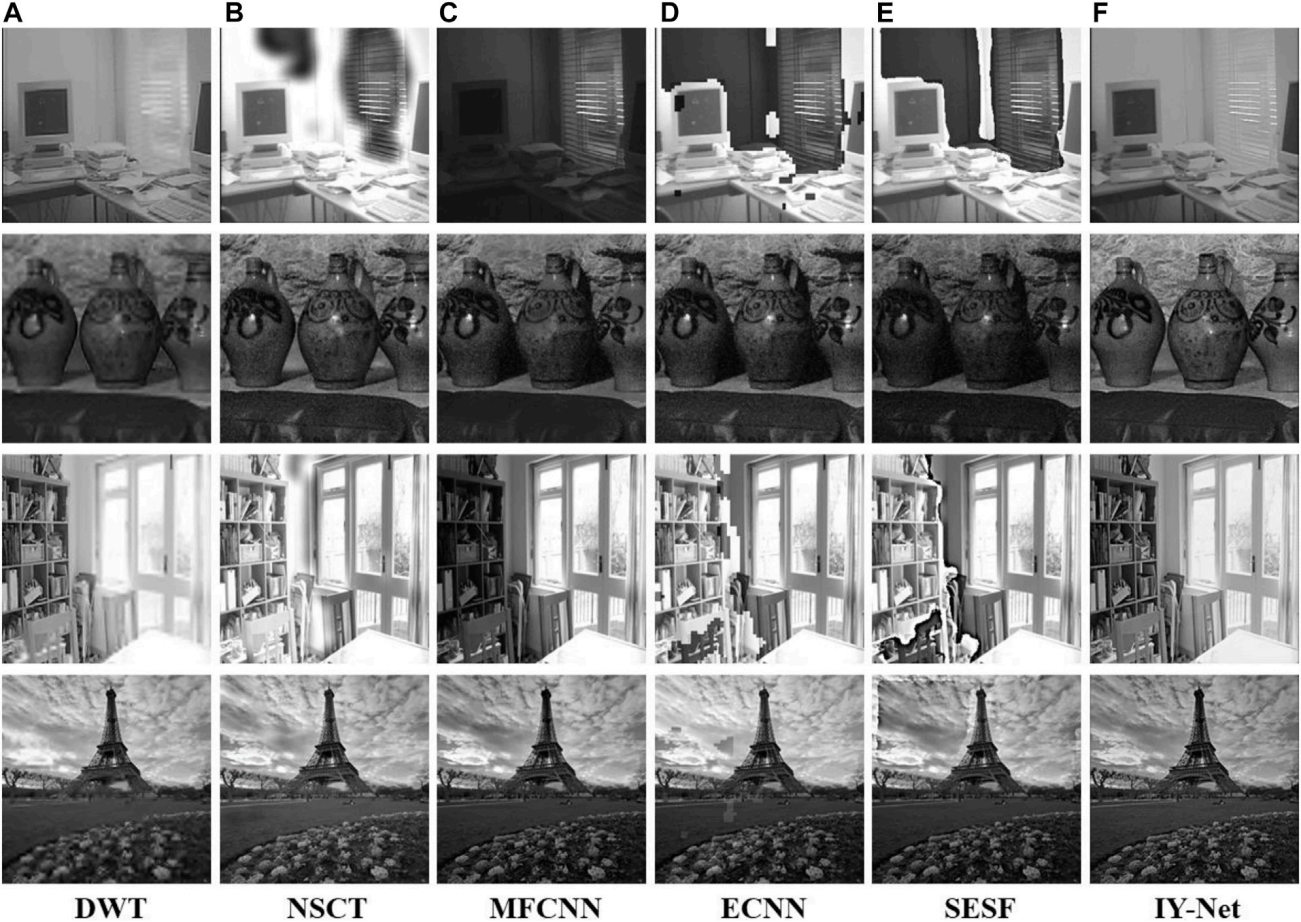
Experiment on 4 pairs of multi-exposure images. **(A)** DWT, **(B)** NSCT, **(C)** MFCNN, **(D)** ECNN, **(E)** SESF, **(F)** IY-Net.

Furthermore, fusion experiments are implemented in multi-exposure images as shown in Supplementary Figure S6 to evaluate the capability of the proposed model. The source “Computer” image is shown in Supplementary Figure S4 (A) and (B), and the two images show high and low exposure images. Supplementary Figure S4 (C)‐(H) shows the fusion results of all algorithms. DWT can keep the source image features, but the region of the curtain is ambiguous. The fused results of NSCT, ECNN, and SESF appear with numerous black spots, and partial details of MFCNN failed to be preserved. In contrast, IY-Net saves the fully-featured texture and achieves great visual effect in multi-exposure image fusion. The results of all the test database fusion are shown in Figure 8 DWT generates blurred textures in some regions. NSCT, MFCNN, and ECNN can effectively respond to fusion in dark environments, but they can lose efficacy for the images with bright information. SESF displays terrible results for different environments, for example, the fused images appear with extensive black spots and distortion of textures. In contrast to these reference algorithms, the proposed model is suitable for multi-exposure image fusion, and the fusion results reflect clearer features and appropriate visual perception.

#### 4.2.5 Remote Sensing Image Fusion

**FIGURE 9:**
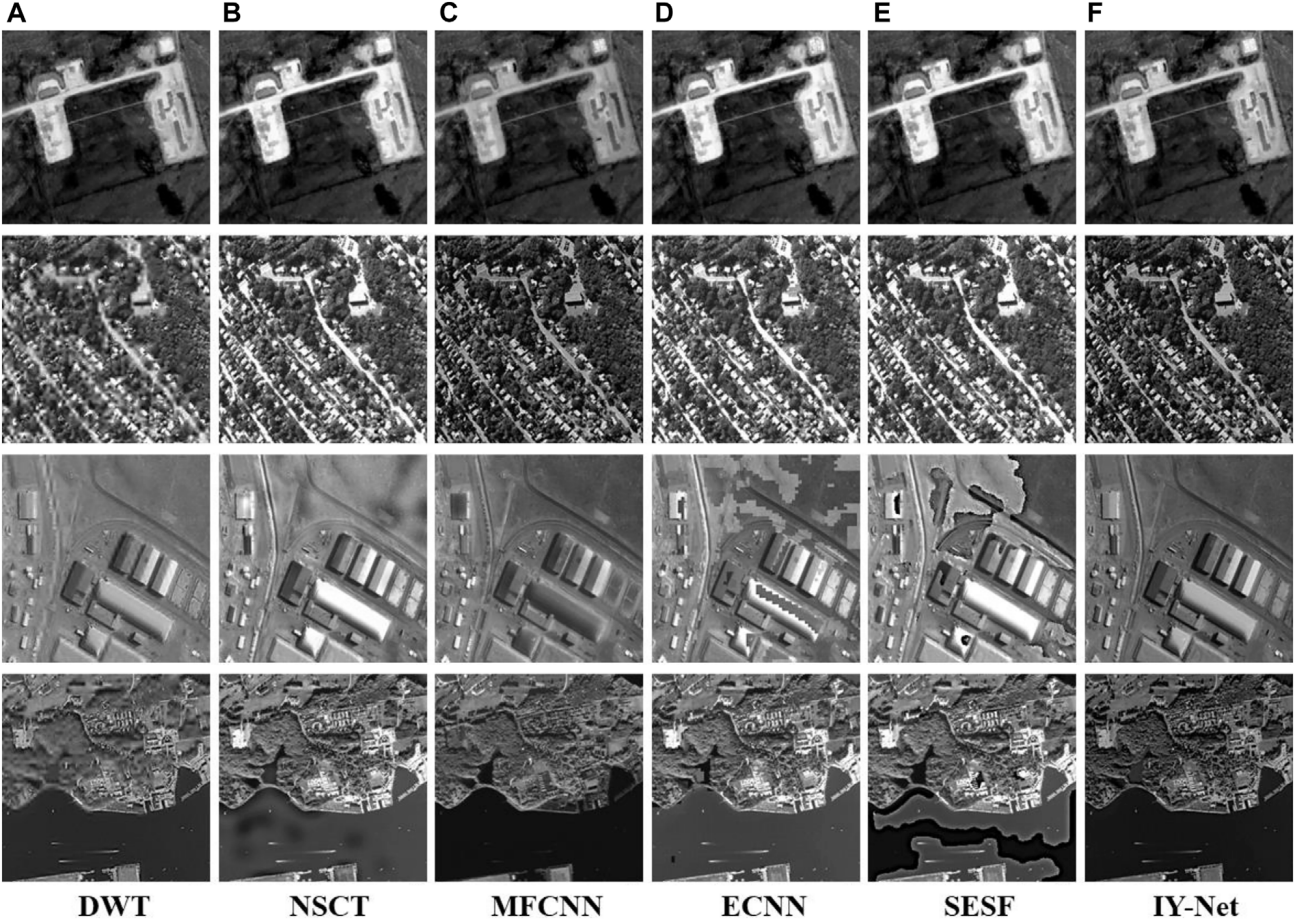
Experiment on 4 pairs of remote sensing images. **(A)** DWT, **(B)** NSCT, **(C)** MFCNN, **(D)** ECNN, **(E)** SESF, **(F)** IY-Net.

Finally, this paper confirms the performance of the proposed model in remote sensing image fusion, and the test dataset is shown in Supplementary Figure S7. The source “Building” images are shown in Supplementary Figures S12 (A) and (B). Supplementary Figures S12 (C)‐(H) show the fusion results of all algorithms. DWT, ECNN, SESF, and NSCT retain most of the detailed features, but some small details are vague. MFCNN and IY-Net can completely detect textures and details, nevertheless, IY-Net has higher contrast and more obvious intensity information than MFCNN. Concerning remote sensing image fusion, IY-Net has a better visual effect. Other fusion results are shown in Figure 9. Experiments reveal that DWT appears to texture distortion, and NSCT has too high contrast and thus obscures some feature information. MFCNN has only a visual perception of single-source image feature information, and ECNN and SESF have a lot of shadows and black spots locally. Obviously, the proposed model has a good visual effect and proper contrast.

### 4.3 Quantitative Comparison and Discussion

Table 1, Table 2, Table 3, Table 4, Table 5 shows the quantitative metrics corresponding to the above multi-modal image fusion results respectively. In these tables, each value represents the average measured value of the dataset, and the best values are bolded. These metrics can be used to fairly and objectively reveal the fusion performance of all the algorithms from an objective perspective combined with subjective vision. As shown in Table 1, IY-Net acquires the optimum Peille metric, which denotes the proposed model is highly correlated with original images compared to these reference algorithms. Although the proposed model failed to yield optimal values for other metrics, the values achieved by the proposed model are acceptable.

**TABLE 1:** Quantitative evaluation results of multi-focus image fusion.

Metrics	DWT	NSCT	MFCNN	ECNN	SESF	IY-Net
SF	21.41603	27.6822	27.5599	**29.5400**	29.4076	22.3491
AG	7.066	9.5068	9.3801	9.6744	**9.7212**	8.2074
IE	7.4132	7.4845	7.4694	**7.4783**	7.4713	7.4591
Q_AB_	0.5012	0.7267	**0.7430**	0.7296	0.7212	0.6880
Peille	0.0076	0.0062	0.0065	0.0064	0.0072	**0.0090**

Bold indicates best values.

**TABLE 2:** Quantitative evaluation results of infrared and visible image fusion.

Metrics	DWT	NSCT	MFCNN	ECNN	SESF	IY-Net
SF	8.1647	12.7831	9.5506	18.3357	**24.9147**	12.5291
AG	3.0915	5.0239	3.6153	5.4813	**7.2602**	4.8389
IE	6.4426	7.166	6.6088	7.1048	**7.3101**	6.8087
Q_AB_	0.328	0.5085	0.4563	**0.5811**	0.5695	0.451
Peille	0.0064	0.0043	**0.0189**	0.0052	0.0129	0.0065

Bold indicates best values.

**TABLE 3:** Quantitative evaluation results of infrared intensity and polarization image fusion.

Metrics	DWT	NSCT	MFCNN	ECNN	SESF	IY-Net
SF	8.8792	13.7983	11.1476	17.0852	**19.5567**	14.2838
AG	3.0686	5.1073	3.9309	5.4514	**6.1073**	5.1259
IE	6.4152	**7.1872**	5.987	6.2169	6.4883	6.9192
Q_AB_	0.3268	0.528	0.5246	0.6061	**0.6156**	0.4627
Peille	0.0073	0.0042	**0.0358**	0.0242	0.0241	0.0049

Bold indicates best values.

**TABLE 4:** Quantitative evaluation results of multi-exposure image fusion.

Metrics	DWT	NSCT	MFCNN	ECNN	SESF	IY-Net
SF	15.5429	23.285	19.9213	29.3432	**30.4046**	22.0839
AG	5.4503	8.7552	6.8542	9.6245	**9.8643**	8.0654
IE	7.1778	7.2096	7.1206	**7.3695**	7.2344	7.2672
Q_AB_	0.4376	0.7668	0.6826	**0.7916**	0.7453	0.7074
Peille	0.0048	0.0027	**0.0103**	0.0036	0.0042	0.0037

Bold indicates best values.

**TABLE 5:** Quantitative evaluation results of remote sensing image fusion.

Metrics	DWT	NSCT	MFCNN	ECNN	SESF	IY-Net
SF	24.9828	31.6788	25.4795	34.3604	**36.9394**	30.3675
AG	9.7509	12.6092	10.1474	12.9351	**13.7295**	11.9796
IE	7.0700	**7.2978**	6.7970	6.8664	6.9975	6.9814
Q_AB_	0.4699	0.6895	0.6557	**0.7131**	0.7049	0.6580
Peille	0.0063	0.0048	**0.0106**	0.0054	0.0069	0.0103

Bold indicates best values.

As can be noticed from the objective metrics in Table 2, SESF acquires the greatest SF, AG and IE values, while ECNN gains the best Q_AB_ value. However, their fusion images present undesirable visual effects as shown in Figure 6. Although the congeneric values of the proposed model are not optimal, they are totally acceptable, especially combining the visual properties of the fusion results. It exhibits that the fusion result with the proposed method is equipped with rich detail and feature information from resource images.

Similar to the objective values in Tables 1 and 2, although the SESF obtained the best values in SF, AG and Q_AB_ in Table 3, it was also mainly caused by unreasonable distortion as shown in Figure 7. There are similar situations in DWT, MFCNN, and ECNN. Even though NSCT can achieve a similar visual effect to the proposed model, the SF, AG, and Peilla values are lower than IY-Net, which indicates that the proposed model has richer image sharpness and edge information, and is highly relevant to the source images.

In Table 4, although the best SF and AG values are attained by SESF and the best Q_AB_ and IE values were yielded by ECNN, it is resulting from the distorted and discordant fusion results as shown in Figure 8. In contrast to these reference algorithms, the proposed model is always stable in the expression of fusion results and the objective metrics are also acceptable, despite IY-Net being unable to highlight the advantages in every metric.

Similar to Table 4, SESF and ECNN in Table 5 also produce abnormal SF, AG and Q_AB_ values caused by partial loss and distortion of image edge information. NSCT achieves a great IE value since some of the fusion results produce redundant feature information. Unlike these reference algorithms, the proposed model can provide excellent visual perception with sound objective values.

In addition to the visual analysis and objective evaluation metrics discuss, the average running time is an important indicator for evaluating algorithm performance. In Table 6, the average running times of all kinds of algorithms are displayed, where the shortest value is bolded. Apparently, the average running time of IY-Net is significantly optimal compared with these reference algorithms, and the proposed neural network model is at least 94% faster than these reference network algorithms. In general, the proposed model has a significant advantage in terms of average running time, compared to these reference algorithms.

**TABLE 6:** Average running time of various algorithms (Time unit: second).

Method	DWT	NSCT	MFCNN	ECNN	SESF	IY-Net
Runtime	0.76	2.025	0.38	0.34	0.31	**0.16**

Bold indicates best values.

Although the reference algorithms yield the best metrics for some modal images, the majority are overestimated due to the incongruous texture features in their fusion results, and they lack generality and stability for different patterns of images. For example, MFCNN, SESF, and ECNN achieved acceptable visual effects only in multi-focus image fusion, and DWT yielded favorable visual effects only in multi-exposure image fusion. As for NSCT, it is also inadequate in generality despite acquiring valuable visual effects in infrared intensity and polarization image fusion and multi-focus image fusion. In contrast, IY-Net can gain reasonable and acceptable quantitative metrics, and it also has significant strengths in the visual effects of multi-modal image fusion, while the computational speed is much faster than these reference algorithms. It reveals that the proposed model has premium generality, stability and rapidity. With the quantitative analysis and running time comparison, it is not difficult to realize that IY-Net achieves outstanding metrics in certain aspects, but there is still much progress to be expected.

## 5 Conclusion

In this paper, a general CNN framework for image fusion is proposed. Compared to current image fusion models, the proposed model has three main advantages: 1) Since it is fully convolutional, the model can be trained end-to-end and without pre-processing. 2) Although the training dataset is comprised of multi-modal images, the fused images not only have outstanding visual effects but also are not impacted by other modal images. 3) Its structure is similar to MSTIF, hence, it has outstanding generality in multi-modal image fusion. To summarize, IY-Net is superior to partial traditional multi-scale algorithms and existing neural network image fusion methods in terms of generality.

The proposed model provides the optimal visual effects compared to these reference algorithms through numerous fusion experiments, but the quantitative metrics are slightly inadequate. There are still several problems to be resolved to get a better-performing image fusion model. Firstly, this paper has a small training dataset, and increasing the large-scale sample may raise the model performance. Secondly, the proposed model consists of only three multiple feature extraction layers, which is relatively simplified, and the efficiency of the model can be enhanced by using a deeper network structure. Thirdly, the loss functions of the model are relatively simple, and the construction of more complex and optimized loss functions may enhance the stability and adaptability of the model.

## Data Availability

The original contributions presented in the study are included in the article/Supplementary Material, further inquiries can be directed to the corresponding author.
