# Supplementary material for: A Two-To-One Deep Learning General Framework for Image Fusion
Source: Front Bioeng Biotechnol. 2022 Jul 14;10:923364. doi: 10.3389/fbioe.2022.923364 (PMC9376963; doi:10.3389/fbioe.2022.923364)
Supplement: Supplementary file 1 [file Presentation1.pdf]

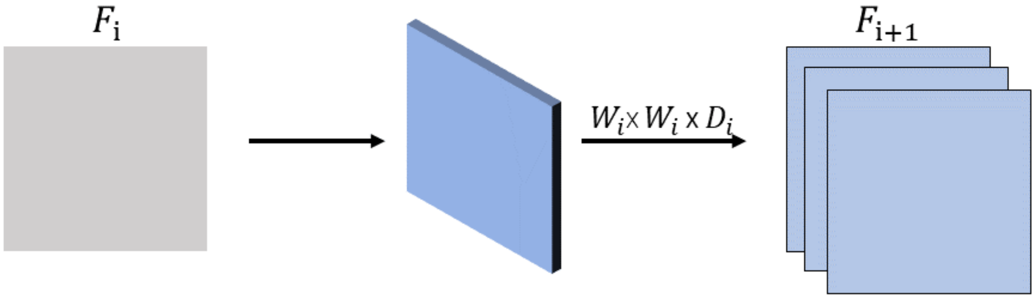

Supplementary Figure 1. General convolutional layer structure. F_i is the input image, F_{i+1} is the output image. W_i is the convolutional kernel size, and D_i is the number of channels.

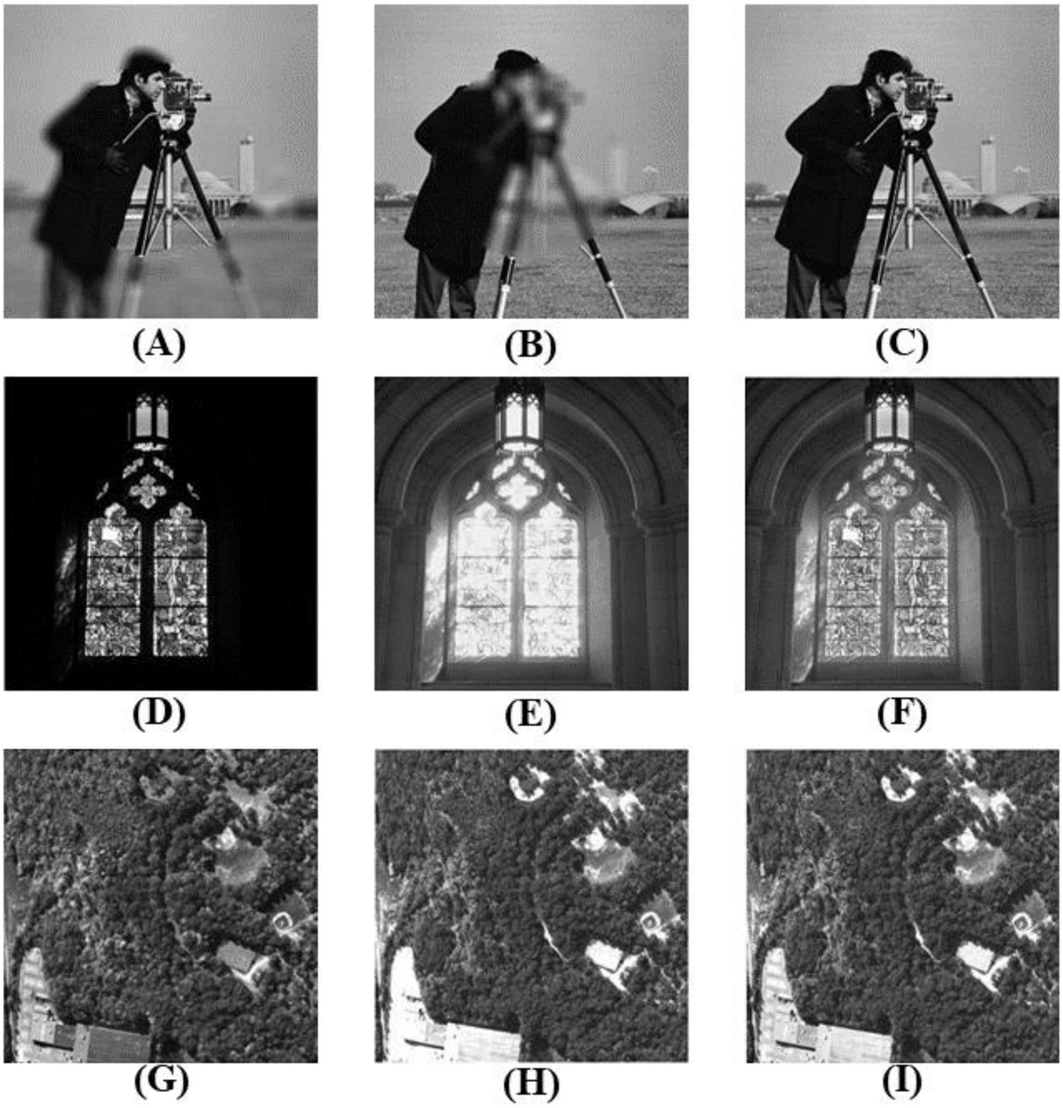

Supplementary Figure 2 Multi-modal image training dataset.

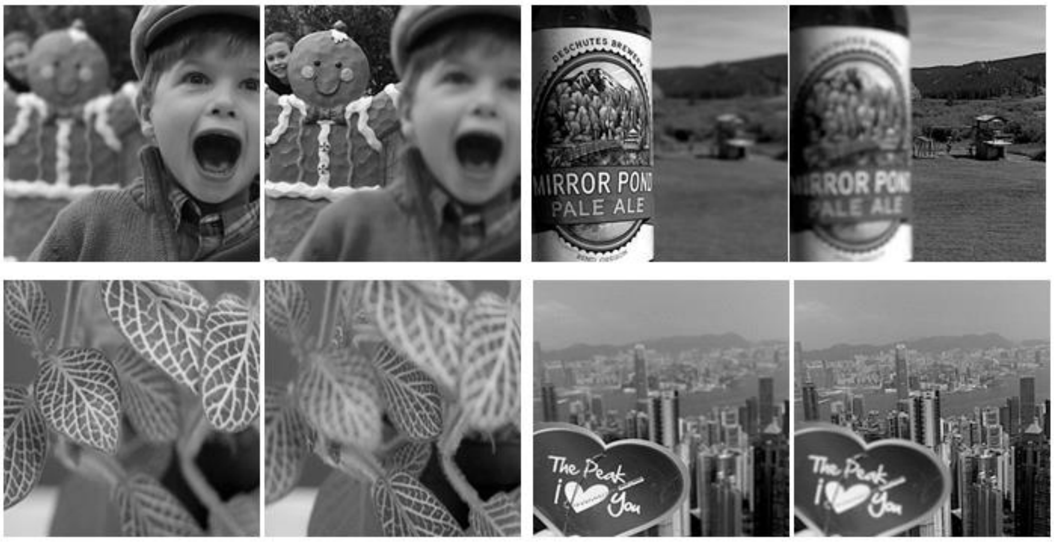

Supplementary Figure 3. Multi-focus image test datasets.

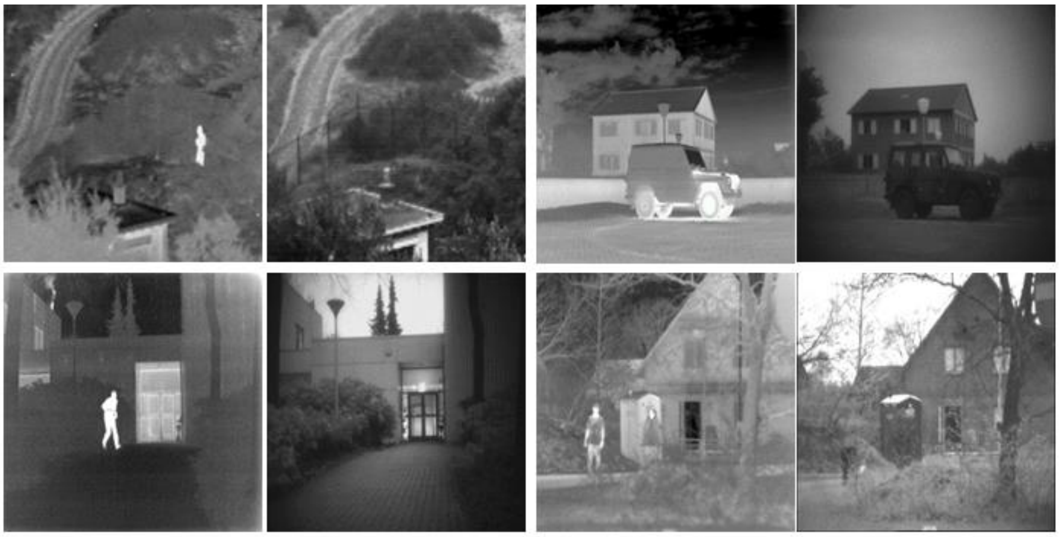

Supplementary Figure 4. Infrared and visible image test datasets.

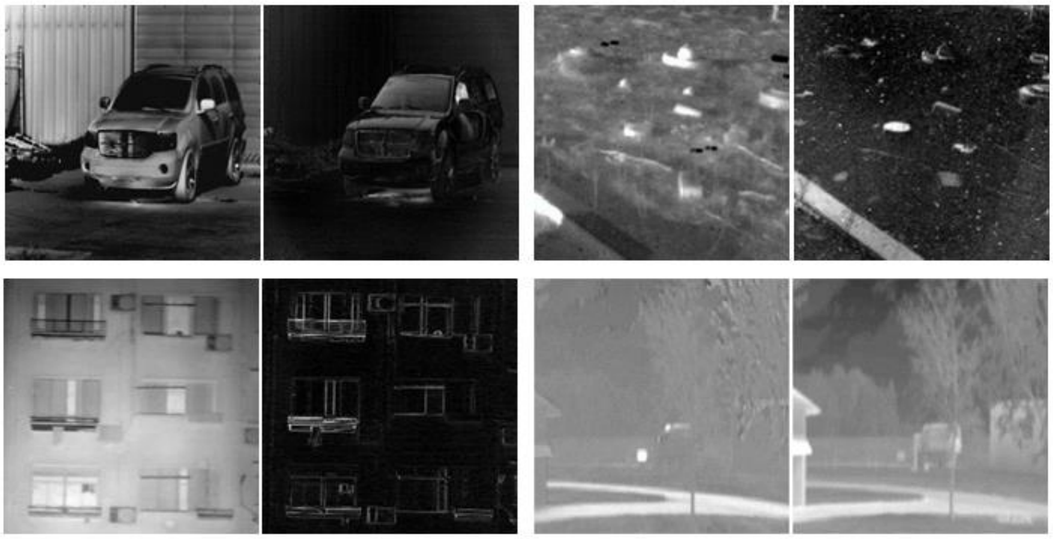

Supplementary Figure 5. Infrared intensity and polarization image test datasets.

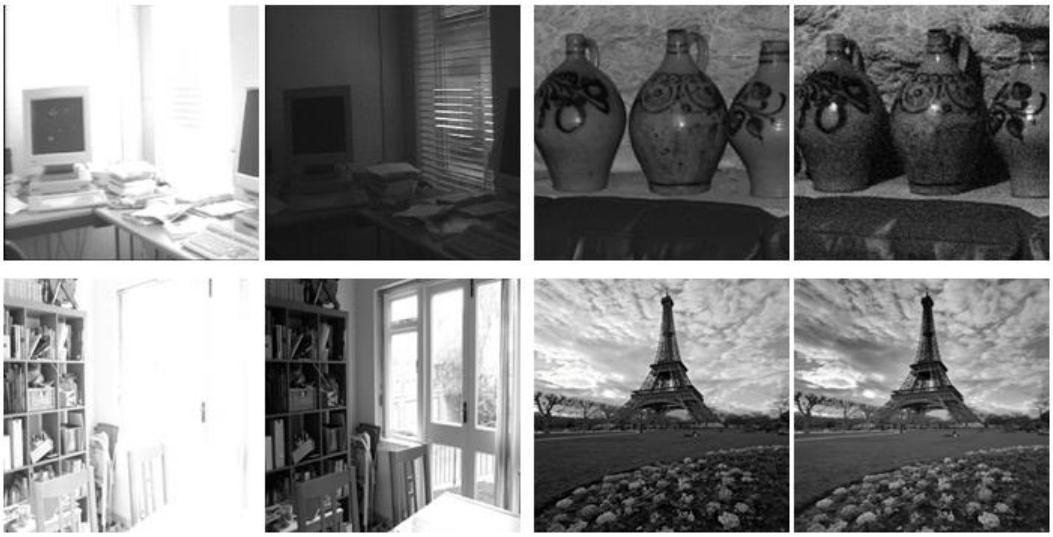

Supplementary Figure 6. Multi-exposure image test datasets.

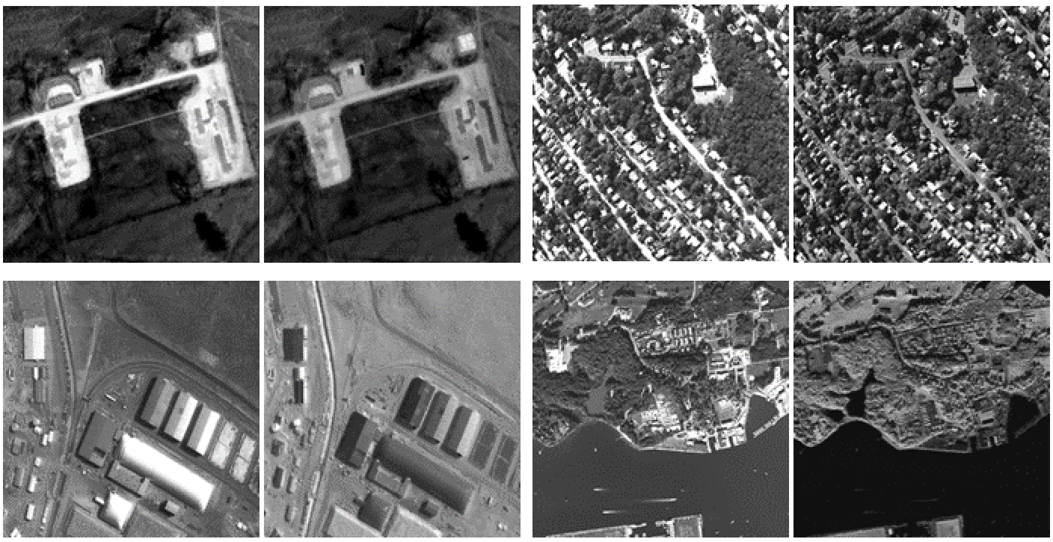

Supplementary Figure 7. Remote sensing image test datasets.

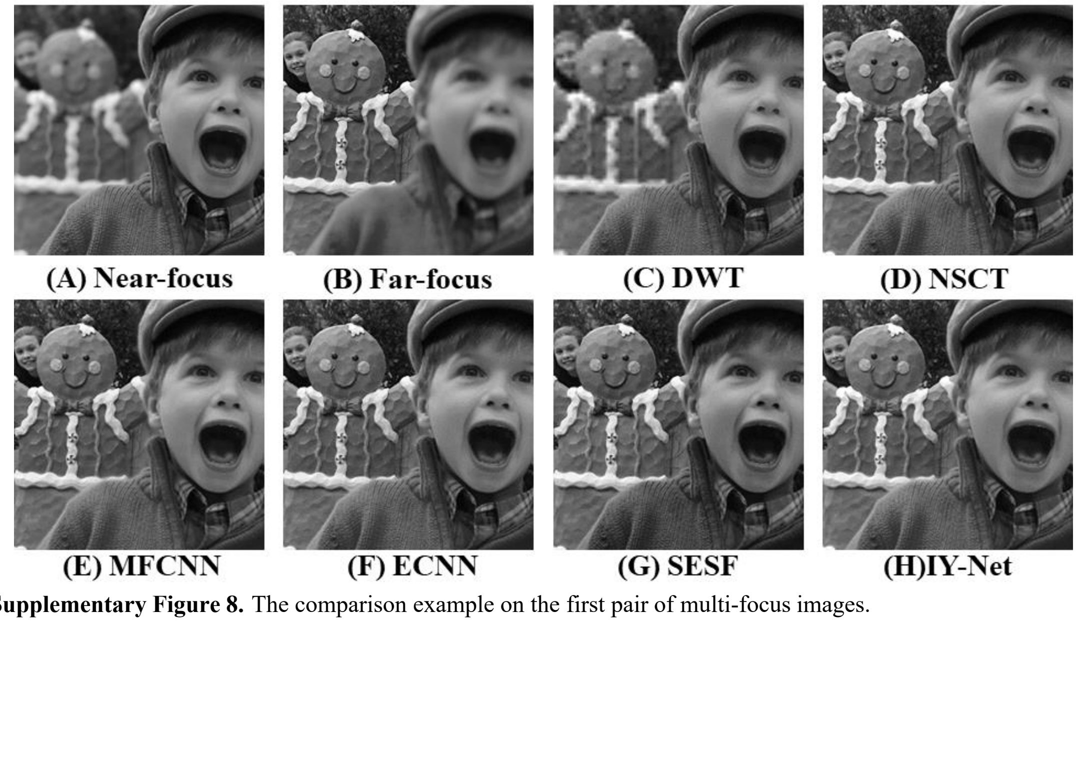

Supplementary Figure 8. The comparison example on the first pair of multi-focus images.

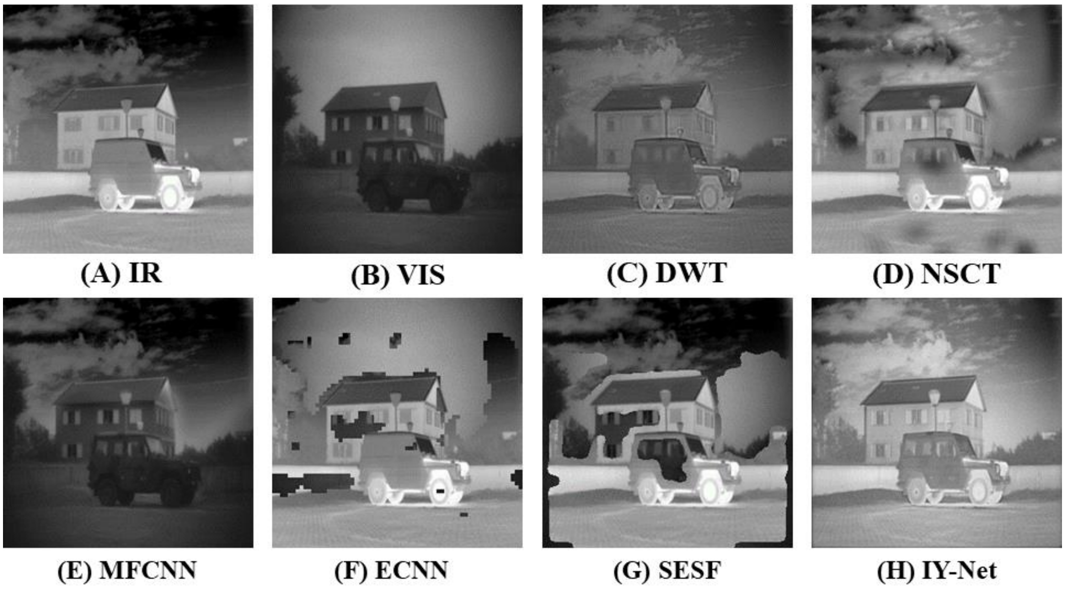

Supplementary Figure 9 The comparison example on the second pair of infrared and visible image.

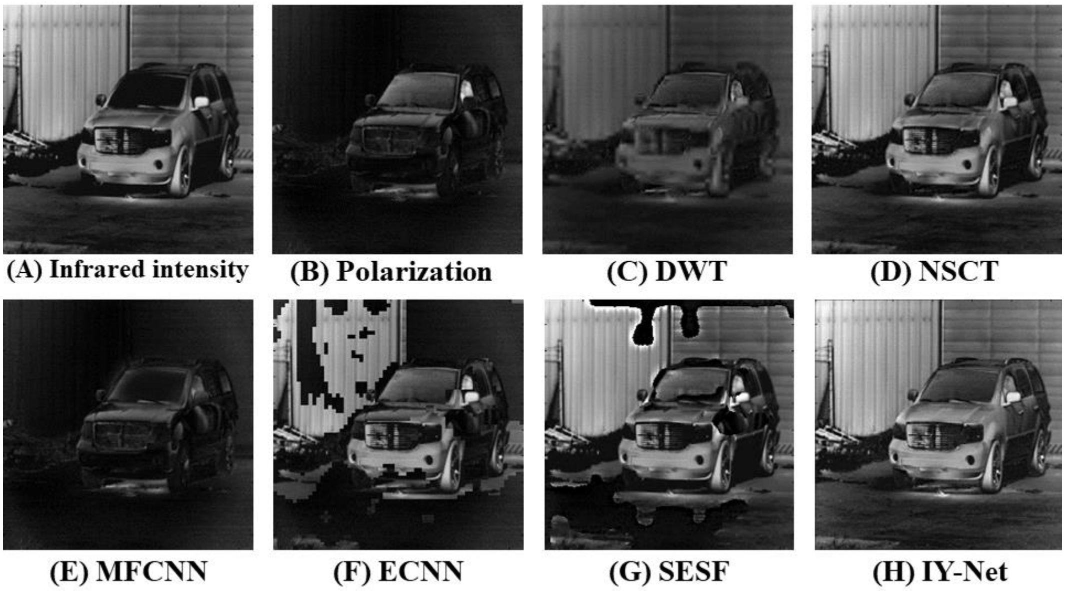

Supplementary Figure 10 The comparison example on the first pair of infrared intensity and polarization images.

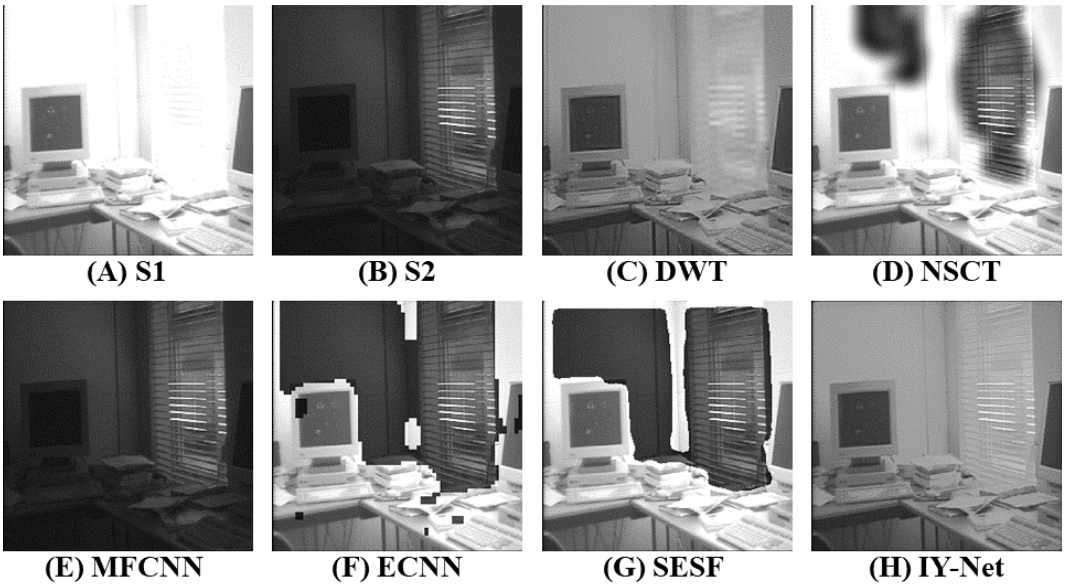

Supplementary Figure 11. The comparison example on the first pair of multi-exposure images.

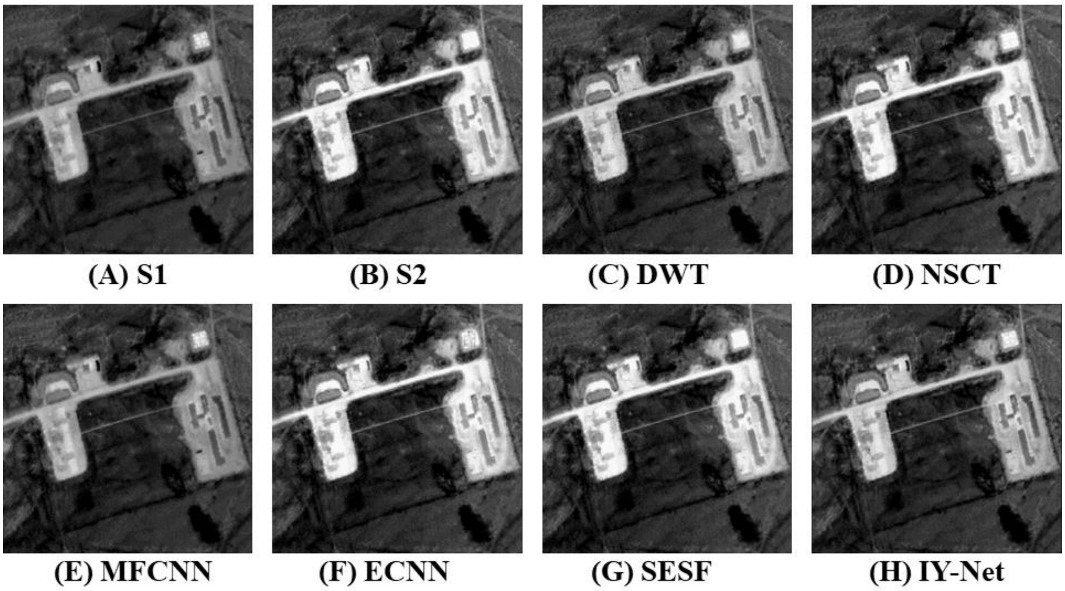

Supplementary Figure 12. The comparison example on the first pair of remote sensing images.
